# Further Insights into The Pathogenic Mechanisms of Haemotropic *Mycoplasma ovis*

**DOI:** 10.21315/tlsr2024.35.3.15

**Published:** 2024-10-07

**Authors:** Paul Bura Thlama, Jesse Faez Firdaus Abdullah, Kamaludeen Juriah, Chung Eric Lim Teik, Che’Amat Azlan, Mohd Lila Mohd Azmi

**Affiliations:** 1 Department of Animal Science and Fisheries, Faculty of Agriculture and Forestry Sciences, Universiti Putra Malaysia Campus Bintulu Sarawak, 97008 Bintulu, Sarawak, Malaysia; 2 Veterinary Teaching Hospital, Faculty of Veterinary Medicine, University of Maiduguri, 600230 Maiduguri Borno, Nigeria; 3 Department of Veterinary Clinical Studies, Faculty of Veterinary Medicine, Universiti Putra Malaysia, 43400 UPM Serdang, Selangor, Malaysia; 4 Institute of Tropical Agriculture and Food Security, Universiti Putra Malaysia, 43400 UPM Serdang, Selangor, Malaysia; 5 Department of Animal Science, Faculty of Agriculture, Universiti Putra Malaysia, 43400 UPM Serdang, Selangor, Malaysia; 6 Department of Veterinary Pathology and Microbiology, Faculty of Veterinary Medicine, Universiti Putra Malaysia, 43400 UPM Serdang, Selangor, Malaysia

**Keywords:** Cellular Pathology, *Mycoplasma ovis*, Pathogenicity, Serum Biomarkers, Patologi Selular, *Mycoplasma ovis*, Patogenik, Biomarker Serum

## Abstract

In this study, we examined the effects of experimental intraperitoneal infection with haemotropic *Mycoplasma ovis* (0.5 mL of blood containing 80% parasitaemia) on selected serum biomarkers and cellular pathology in mice. After infection, *M. ovis* cells appeared in the blood films within one week. A dose-dependent peak of parasitemia was observed during the 3^rd^-week post-infection (pi), with a significant decrease in mean PCV between treatment versus control group at week 3 (*t*_14_ = −3.693, *P <* 0.02), week 5 (*t*_14_ = −2.096, *P* = 0.055), and week 7 (*t*_14_ = −4.329, *P =* 0.001). There was a significantly (*t*_8_ = −2.330, *P* = 0.048) lower serum oestrogen in treatment (10.38 ± 5.07) than control (17.43 ± 4.48), while serum progesterone was significantly (*t*_8_ = 5.415, *P* = 0.001) increased in treatment (27.37 ± 2.17) than control (15.92 ± 4.20). Serum haptoglobin was significantly (*t*_8_ = 8.525, *P* < 0.01) lower in treatment (8.72 ± 1.49) than control (18.16 ± 1.98) while the SAA was significantly (*t*_8_ = 3.362, *P* = 0.01) higher in treatment (16.79 ± 2.71) than control (11.59 ± 2.15). Prominent lesions observed in the ovary include degeneration, necrosis, vacuolation, and hypertrophy of the lutein cells in corpora lutea. In the lymph nodes, diffused cellular hyperplasia of the lymphoid tissue in the cortex. In the liver, degeneration and necrosis accompanied by leucocytic cellular infiltration and Kupffer cell proliferation within the sinusoids. There were diffused leucocytic infiltrations and proliferative lesions in the glomerulus of the kidneys. The disturbance in progesterone and ovarian pathology highlights the potential role of haemotropic *M. ovis* in reproductive disorders. The observed changes in biomarkers and cellular reactions following *M. ovis* infection in the mouse may be further advanced in sheep and goats.

## Abstract (fr)

**Abstrak:**

Dalam kajian ini, kami mengkaji kesan jangkitan intraperitoneal eksperimen dengan *Mycoplasma ovis* hemotropik (0.5 mL darah yang mengandungi 80% parasitemia) pada biomarker serum terpilih dan patologi selular pada tikus. Selepas jangkitan, sel *M. ovis* muncul dalam filem darah dalam masa satu minggu. Kemuncak parasitemia yang bergantung kepada dos diperhatikan semasa minggu ke-3 selepas jangkitan (pi), dengan penurunan ketara dalam purata PCV antara rawatan berbanding kumpulan kawalan pada minggu ke-3 (*t*_14_ = −3.693, *P* < 0.02), minggu ke-5 (*t*_14_ = −2.096, *P* = 0.055), dan minggu 7 (*t*_14_ = −4.329, *P* = 0.001). Terdapat estrogen serum yang jauh lebih rendah (*t*_8_ = −2.330, *P* = 0.048) dalam rawatan (10.38 ± 5.07) daripada kawalan (17.43 ± 4.48), manakala progesteron serum adalah ketara (*t*_8_ = 5.415, *P* = 0.001) meningkat dalam rawatan (27.37 ± 2.17) daripada kawalan (15.92 ± 4.20). Serum haptoglobin adalah ketara (*t*_8_ = 8.525, *P* < 0.01) lebih rendah dalam rawatan (8.72 ± 1.49) daripada kawalan (18.16 ± 1.98) manakala SAA adalah ketara (*t*_8_ = 3.362, *P* = 0.01) lebih tinggi dalam rawatan (16.79 ± 2.71) daripada kawalan (11.59 ± 2.15). Lesi yang menonjol yang diperhatikan dalam ovari termasuk degenerasi, nekrosis, vakuolasi dan hipertrofi sel lutein dalam corpora lutea. Di kelenjar getah bening, hiperplasia selular meresap tisu limfoid di korteks. Di dalam hati, degenerasi dan nekrosis disertai dengan penyusupan selular leukositik dan percambahan sel Kupffer dalam sinusoid. Terdapat penyusupan leukosit yang meresap dan lesi proliferatif dalam glomerulus buah pinggang. Gangguan dalam patologi progesteron dan ovari menyerlahkan potensi peranan hemotropik *M. ovis* dalam gangguan pembiakan. Perubahan yang diperhatikan dalam biomarker dan tindak balas selular berikutan jangkitan *M. ovis* pada tikus mungkin lebih maju pada biri-biri dan kambing.

**Highlights**
- Experimental *Mycoplasma ovis* infection in mice produced significant vascular changes, cellular degeneration, necrosis and hypertrophy of ovarian lutein cells accompanied by a disturbance in female reproductive hormones.
- The dysregulation of progesterone and ovarian pathology observed here are novel findings that elucidate the potential mechanism of reproductive disorders associated with haemotropic *M. ovis* under field conditions.
- The ovarian pathology and immunological reactions seen in the liver, spleen, lymphnodes, and kidneys could potentially influence folliculogenesis and drive hormonal imbalances.

## INTRODUCTION

Haemotropic *M. ovis* is an important cause of anaemia, retarded growth and undesirable production outcomes (Hornok *et al*. 2012; Rani *et al*. 2018; Jesse *et al*. 2020), including poor reproductive performance, decreased milk yield in dairy cows (Messick 2004), and the incidence of abortion in sheep flocks (Urie *et al*. 2019). The presence of parasites in aborted foetuses from infected cows hinted the potential involvement of the reproductive system (Hornok *et al*. 2011). Moreover, recent reports on neonatal haemoplasma infection in calves further strengthened evidence suggesting transplacental transmission of infection in cows (Girotto-Soares *et al*. 2016).

Acute-phase proteins (APPs) and cytokines are presently used as biochemical markers to support the clinical diagnosis of many diseases (Othman *et al*. 2016; Raynes 1994; Chung *et al*. 2019). The proinflammatory cytokines such as interleukin (IL)-1, IL-2, tumour necrosis factor (TNF)-α, and interferon-gamma (IFN-γ) activate cellular defences to pathogens during the acute phase response (APR) (Horuk 1994). A significant component of the acute inflammatory stimulus produced by cytokine release is the activation of hepatic biosynthesis of APPs (Uhlar & Whitehead 1999), such as serum amyloid A (SAA), haptoglobin (Hp), lipopolysaccharide-binding protein (LBP) and α-1-acid glycoprotein (AGP) (Eckersall & Bell 2010). The SAA and Hp molecules are recognised as major APPs that serve as binding proteins and immune modulators (Ceciliani *et al*. 2012), whose concentrations may increase dramatically during the APR (Jain *et al*. 2011). The liver synthesises Hp in response to specific chemokines (Raynes 1994). The Hp molecule participates in scavenging free haemoglobin in the blood, regulating innate immunity in white blood cells, exerting direct bacteriostatic effects and chaperone activity (Ceciliani *et al*. 2012). The SAA molecule, produced by the liver in response to TNF-α and IL-6 (Uhlar & Whitehead 1999), participates in opsonisation, preventing cholesterol aggregation at the site of inflammation and modulating the innate immune response during the APR (Jain *et al*. 2011). Therefore, these markers were selected to evaluate their potential role in the pathogenesis of experimental haemotropic *M. ovis* infection. The mouse model was used in this study because murine species have been used to study various mechanisms of haemoplasma infection (Messick 2004; Rani *et al*. 2018; Hornok *et al*. 2012; Paul *et al*. 2020).

The application of microscopy in the current diagnosis of haemotropic *M. ovis* yields low sensitivity. Similarly, serology produces nonspecific products, while PCR is expensive and requires expertise (Neimark *et al*. 2004; Faraj & Kamal 2017). Since recent studies have recorded success in the assay of serum biomarkers as potential early markers of infections (Jain *et al*. 2011; Musaya *et al*. 2015; Chung *et al*. 2019), we explored serum Hp and SAA as potential markers for the clinical diagnosis of *M. ovis*. Furthermore, haemoplasma infections are known to cause decreased milk yield and abortion in domestic animals, but their impact on reproductive physiology remains unresolved. In this study, we examined the effects of experimental intraperitoneal infection of haemotropic *M. ovis* on selected serum biomarkers and cellular pathology in the mouse model. It was hypothesised that the average observations on the PCV, serum biomarkers and cellular lesions in the *M. ovis* treatment and control group are the same.

## MATERIALS AND METHODS

### Ethics Approval

The experimental and laboratory protocols outlined in this study were approved by the Institutional Animal Care and Use Committee (IACUC), Universiti Putra Malaysia (UPM/IACUC/AUP-R041/2019). The study protocols also comply with the international guidelines for Ethical Conduct in the Care and Use of Laboratory Animals in Biomedical Research.

### Haemotropic *Mycoplasma ovis* Inoculum

Uncharacterised wild strain of *M. ovis* obtained from infected sheep under a semi-intensive management system was used for preparing the inoculum (Paul *et al*. 2021). Two healthy adult guinea pigs were initially inoculated intraperitoneally with heavily parasitised blood samples obtained from haemoplasma-infected sheep to further propagate *M. ovis* infection for the subsequent infection of the treatment group. The infection was monitored by microscopic examination of blood smears until the peak of parasitaemia (day 21 pi). The parasite score of the inoculum (80% infected cells) used to inoculate the treatment group was determined by the examination of 10 microscopic fields of Giemsa-stained blood smears at the peak of parasitaemia (Gulland *et al*. 1987).

### Experimental Design

A controlled experiment was designed to investigate the null hypothesis “that the average observations on the PCV, serum biomarkers, and cellular lesions in the *M. ovis* treatment and control group are the same”. The sample size (*n*) was calculated using G*Power software based on a 90% effect size, 95% CI, 5% level of significance and a two-sided *t*-test. Sixteen healthy adult (10 weeks old) female Institute of Cancer Research (ICR) mice strain (33.5 ± 0.2 g) were randomly assigned to the control (*n* = 8) or treatment (*n* = 8). The mice were acclimatised for 14 days and maintained at a temperature of 22 ± 1°C under 12-hour light/darkness cycles in separate plastic cages. Feeding was done manually using commercial pelletised mouse chow (Golden Coin Feeds, Serdang BHD), and tap water was given *ad libitum* throughout the trial period. Oestrus synchronisation was performed to bring all the mice in the reproductive phase using 0.5 μg/mouse of cloprostenol (PGF2α 250 μg/mL) and a 3 μg of progesterone injection given at week 1 (Pallares & Gonzalez-Bulnes 2009). In week 2, we infected the treatment group with 0.5 mL of inoculum (80% infected cells) by intraperitoneal injection and the control group was given 0.5 mL of sterile physiological buffered saline (PBS) pH 7.0 by intraperitoneal injection. The progression of infection was monitored through weekly examination of parasitaemia and haematocrit from tail blood. All mice in the control and treatment group were euthanised by anaesthesia using a combination of 100 mg/kg ketamine and 10 mg/kg xylazine on day 56 pi. Immediately after euthanasia, blood was drained from the heart using a 25-gauge needle and syringe.

### Evaluation of PCV and Parasitaemia in Mice

The PCV was determined using the microhaematocrit centrifugation technique (Grindem, 2011). Smears of whole blood prepared on glass slides (75 mm × 25 mm) were fixed with methyl alcohol and stained with 10% Giemsa solution. Ten high-power fields were examined on a thin section of each blood smear under bright-field microscopy using an oil immersion objective lens (×100) to detect and count the number of infected cells (*n*) per 1,000 erythrocytes during infection (Gulland *et al*. 1987; Jesse, Jazid, *et al*. 2015).

### Enzyme-Linked Immunosorbent Assays (ELISA)

Serum was extracted from clotted blood by centrifugation at 3,000 RPM for 10 min at 6°C (Eppendorf, Hamburg, Germany). The serum samples were stored at −20°C until used for the ELISA tests. Commercial sandwich ELISA test kits were used for the quantitative detection of serum amyloid A (catalogue number: E0372Mo; LOT 20190008) and oestrogen (catalogue number: E1480Mo; LOT 20190008) while competitive ELISA test kits were used for quantifying the serum haptoglobin (catalogue number: EA0023Mo, LOT 20190008) and progesterone (catalogue number: EA0016Mo, LOT 2019008). All assay procedures were performed according to the manufacturer’s instructions (Bioassay Laboratories, China), and the optical densities of the assays were determined at 450 nm using an ELISA microplate reader (Sunrise^®^, Tecan AG, Switzerland).

### Gross and Histopathological Evaluations

The visceral organs were initially examined *in situ* at postmortem to record any changes in size, shape, colour and consistency. The ovaries, liver, kidney, spleen and lymph nodes were harvested and fixed in 10% buffered formalin. After fixation for two weeks, the tissues were subjected to routine paraffin embedding, microtome sectioning, staining with haematoxylin-eosin (H&E) and mounted with xylene dibutyl phthalate (DPX). A minimum of six microscopic fields were examined per slide of tissue sections at 400× magnification of a compound microscope to assess cellular changes such as degeneration, necrosis, infiltrations, oedema, congestion and haemorrhages (Khaleel *et al*. 2014).

### Statistical Analyses

The optical densities of ELISA microplates were quantitatively analysed using a 4 four-parameter logistic curve fit to calculate the concentrations of cytokines, APPs and reproductive hormones in the serum samples (https://myassays.com/). An independent-sample *t*-test was conducted using SPSS software version 25.0 to determine whether there is a significant difference between the treatment and control groups regarding the packed cell volume, oestrogen, progesterone, Hp and SAA. The mean differences between the treatment and control groups were considered significant at *P* ≤ 0.05.

## RESULTS

### Trends in Parasitaemia and PCV of Mice Responding to *Mycoplasma ovis* Infection

**Figure 1:**
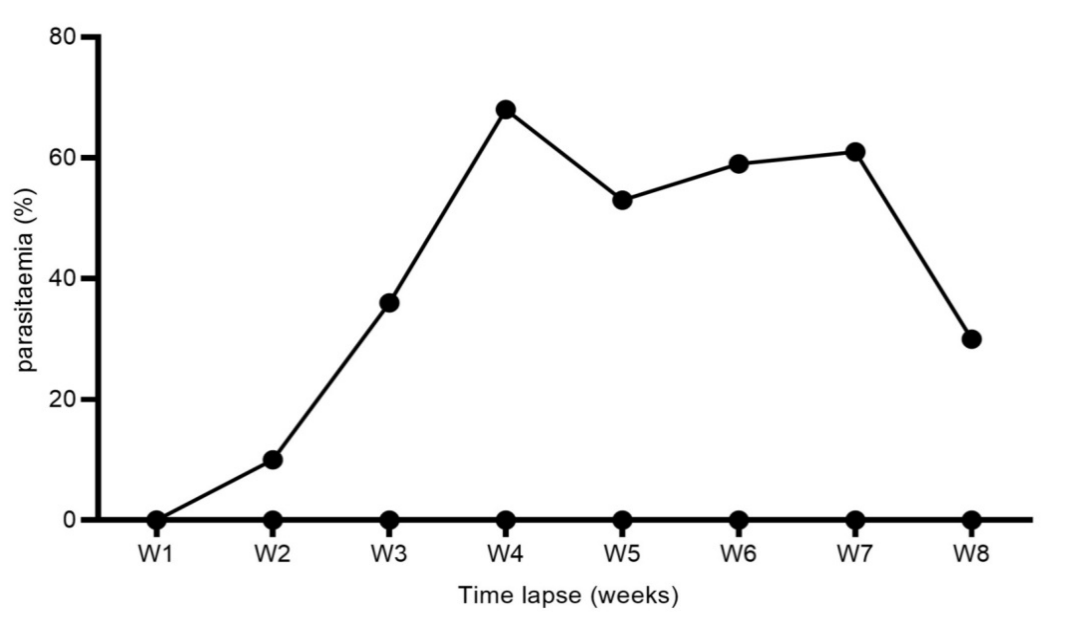
Observed pattern of parasitaemia in mouse responding to *M. ovis* infection.

**Figure 2:**
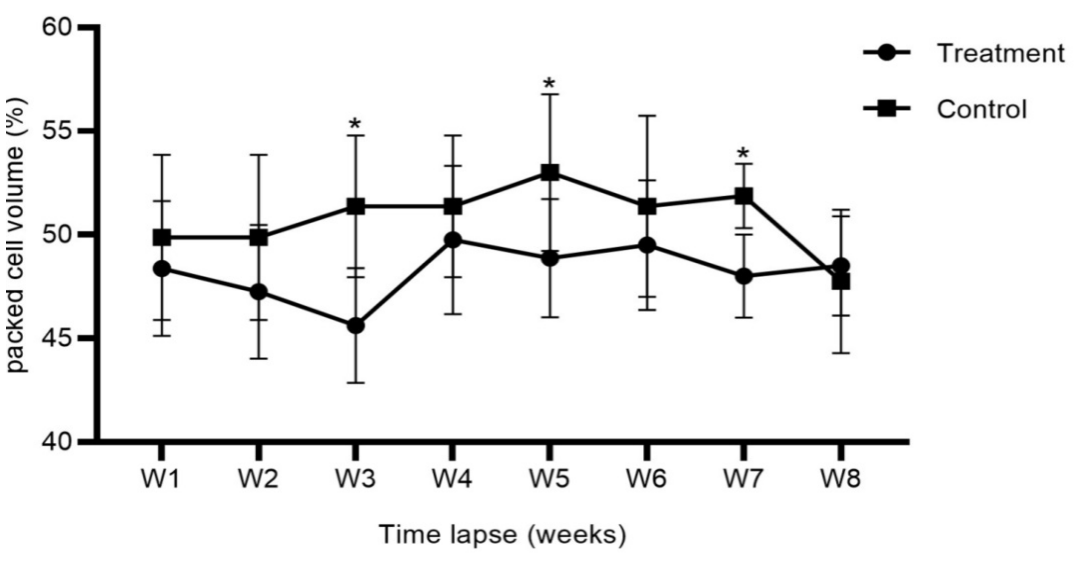
Mean PCV values of mice responding to *M. ovis* infection.

*M. ovis* was first detected in the blood one week post-infection (week 2) with a mean parasitaemia of 10% (5–15). The severity of infection increased sharply to reach its peak in week 4, 68% (56–85) and decreased to 30% (19–45) in week 8 (Fig. 1). The results of an independent samples *t*-test indicate a significant decrease in mean PCV between the treatment versus control group at week 3 (*t*_14_ = −3.693, *P <* 0.02), week 5 (*t*_14_ = −2.096, *P* = 0.055), and week 7 (*t*_14_ = −4.329, *P =* 0.001). The magnitude of difference in mean PCV between the treatment and control group was highest in week 3 pi (−5.75, 95% CI: −9.10 to −2.40), followed by week 5 (−4.13, 95% CI: −8.35 to 0.10), and week 7 (−3.88, 95% CI: −5.79 to 2.20) (Fig. 2).

### Changes in Selected Serum Biomarkers of Mice Responding to *Mycoplasma ovis* Infection

The results of an independent samples *t*-test indicate a significantly (*t*_8_ = 2.330, *P* = 0.048) lower serum oestrogen in the treatment (10.3766 ± 5.07) than control (17.43 ± 4.48), and the magnitude of difference in oestrogen between the groups was 7.05 (95% CI = 0.72 – 14.03). Conversely, there was a significantly (*t*_8_ = 5.415, *P* = 0.001) higher mean serum progesterone in the treatment (27.37 ± 2.17) than control (15.92 ± 4.20), and the magnitude of difference in progesterone between the groups was 11.45 (95% CI = 6.58–16.33). The concentration of serum haptoglobin was significantly (*t*_8_ = 8.525, *P* < 0.01) lower in the treatment (8.72 ± 1.49) than control (18.16 ± 1.98), and the magnitude of difference in haptoglobin between the groups was 9.44 (95% CI = 6.89–11.99). However, there was a significantly (*t*_8_ = 3.362, *P* = 0.01) higher SAA in the treatment (16.79 ± 2.71) than in the control (11.59 ± 2.15), and the magnitude of difference in SAA between the groups was 5.21 (95% CI = 1.63–8.78) (Table 1).

### Gross and Histopathological Changes in Mice Responding to *M. ovis* Infection

**Figure 3:**
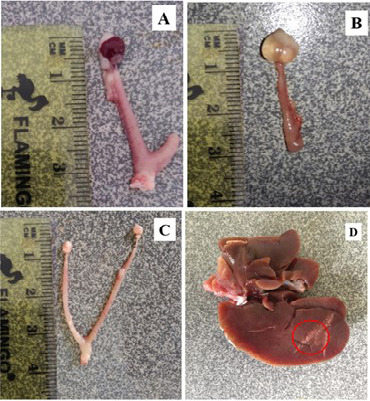
Photomicrographs of postmortem lesions observed in mouse responding to *M. ovis* infection. (A) haemorrhage on the ovary of treatment group, (B) fluid accumulation on the ovary of treatment group, (C) normal reproductive tract in the control group, and (D) grey areas of necrosis on the liver of treatment group.

**Figure 4:**
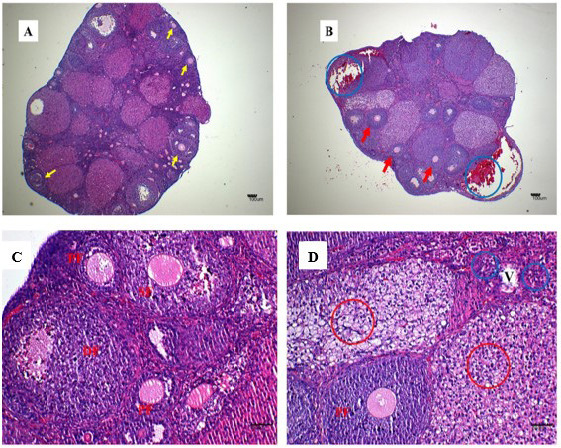
Photomicrograph of the whole ovary in a mouse responding to *M. ovis* infection. (A) control group showing numerous follicles (yellow arrows), (B) treatment group showing haemorrhages (blue circles), fewer follicles (red arrows) and active corpora lutea (CL), (C) control group showing primary follicles (PF), the secondary follicle (SF), and a degenerate follicle (DF), and (D) treatment group showing primary follicle (PF), vacuolation and hypertrophy of the lutein cells (red circles) in adjoining corpora lutea and leucocytic infiltration (blue circle) around a blood vessel (V) (400x). Haematoxylin and Eosin stain.

**Figure 5:**
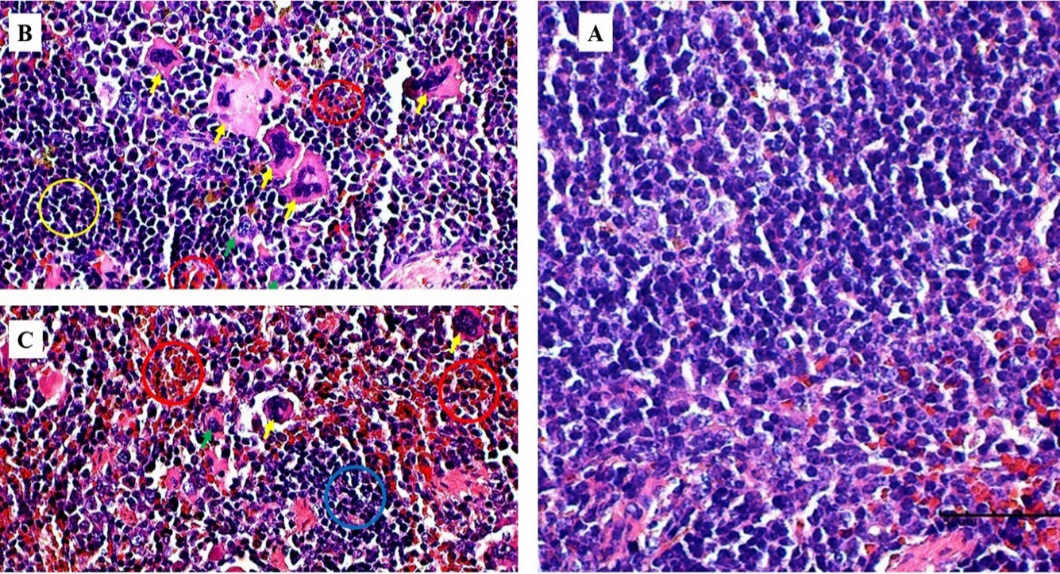
(A) control group showing normal appearance of lymphoid tissue in the splenic red pulp; (B) treatment group showing several neutrophils (yellow arrows) and macrophage (green arrow), mild haemorrhage (red circle) and hypercellularity (yellow circle); and (C) treatment group showing severe haemorrhage (red circle), hypercellularity (blue circle), infiltration of macrophages (green arrow) and neutrophils (yellow arrow) in the red pulp of the spleen.

**Figure 6:**
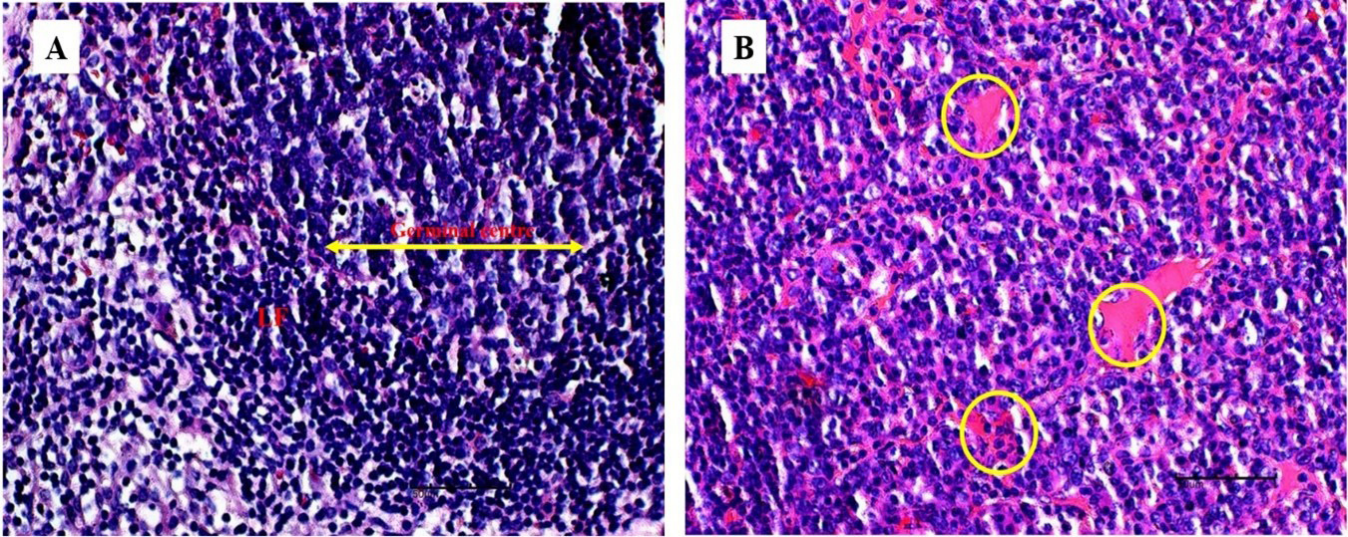
(A) control group showing a germinal centre (yellow arrow) within a lymphoid follicle (LF); (B) treatment group mouse showing severe congestion (yellow circles) and diffused cellular hyperplasia of the lymphoid tissue in the femoral lymph node cortex.

**Figure 7:**
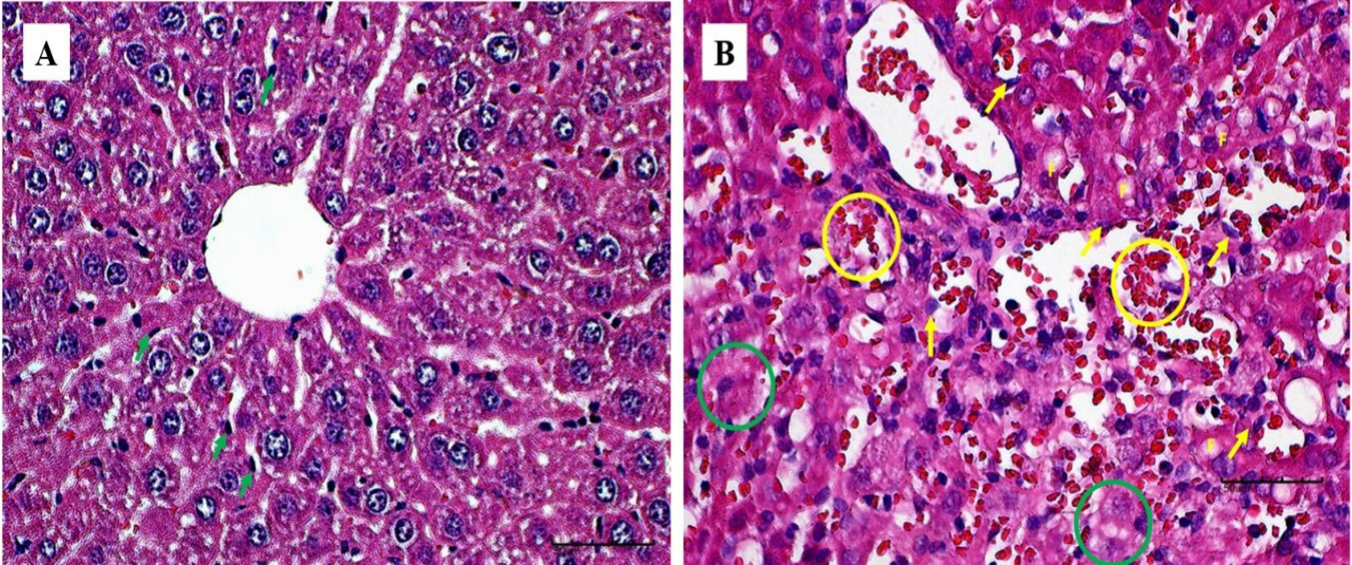
(A) control group showing normal population and size of Kupffer cells (green arrows) in the liver parenchyma; and (B) treatment group showing increased number and change in the shape and size of Kupffer cells due to phagocytosis of infected erythrocytes (yellow arrows), severe congestion of sinusoids (yellow circle), severe and diffused necrosis of hepatocytes (green circles), severe leucocytic infiltration and fatty change (F).

**Figure 8:**
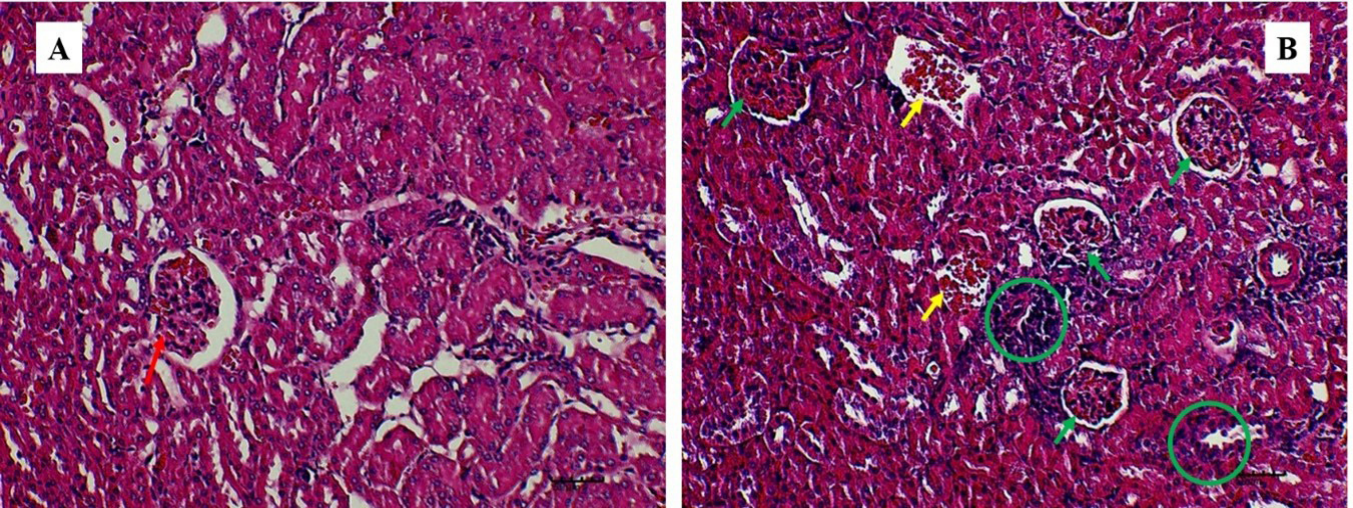
(A) The renal cortex in control group showing normal glomerular tuft (red arrow); (B) treatment group with a proliferative lesion in the glomerulus (green arrows), severe leucocytic infiltration (green circles) and severe congestion of renal veins (yellow arrows) (400×). Haematoxylin and Eosin stain.

The gross lesions observed in the treatment group include a plug of coagulated blood covering the right ovary (Fig. 3A), transparent fluid-filled thin membrane-bound cyst on the ovary (Fig. 3B), oedematous fallopian tubes (Fig. 3C), and an enlarged friable liver with a greyish white zone (red circle) on the visceral surface of the left lobe (Fig. 3D). Microscopically, the predominant lesions in the ovaries include significant vascular changes (congestion and haemorrhages), cellular changes (degeneration, necrosis, vacuolation, and hypertrophy of the lutein cells in corpora lutea) (Figs. 4B–4D). The spleen also showed significant vascular haemorrhage and congestion accompanied by cellular infiltration of macrophages and neutrophils in the red pulp in the treatment group (Figs. 5B and 5C). In the lymph nodes, there was severe generalised vascular congestion and diffused cellular hyperplasia of the lymphoid tissue in the cortex of the treatment group (Fig. 6B). In the liver, there were significant vascular congestions and haemorrhages, cellular degeneration and necrosis accompanied by leucocytic cellular infiltration and Kupffer cell proliferation within the sinusoids in the treatment group (Fig. 7B). In the kidneys, there was significant vascular congestion, haemorrhages and evidence of fluid accumulation, cellular degenerations, leucocytic infiltrations, and a proliferative lesion in the glomerulus in the treatment group (Fig. 8B).

## DISCUSSION

This study investigated the trends of parasitaemia and haematocrit and the responses of APPs, as well as primary female reproductive hormones, during experimental infection with haemotropic *M. ovis* in the murine model. It was hypothesised that the average observations on the PCV, serum biomarkers, and cellular lesions in the *M. ovis* treatment and control group are the same. However, this study showed a significant change in haematocrit during the peak of parasitaemia in experimental *M. ovis* infection, as seen in sheep (Sutton 1979). But, contrary to Foogie and Nisbet (1964), who reported clinical anaemia in experimental *Eperythrozoon ovis* infection of sheep, the present study has found reduced haematocrit without any evidence of clinical anaemia in infected mice. The discrepancies in results between these studies may be attributed to differences in the animal models and experimental conditions. The parasitaemia decreased beyond 28 days pi, and haematocrit regained progressively to attain nearly pre-treatment levels at day 56. Although there was a rapid onset of infection with *M. ovis* appearing in Giemsa-stained blood films 14 days post-infection, causing a significant impact on the haematocrit, recovery occurred spontaneously in the course of the disease, suggesting that haemotropic *M. ovis* infection is self-limiting-in-apparently-healthy individuals. The observed trends in parasitaemia and packed cell volume in the present study are similar to those of *E. ovis* infection in sheep (Sutton & Jolly 1973).

Concerning the serum values of Hp, SAA, oestrogen, and progesterone, different observations were recorded among the treatment and control groups, which is contrary to the null hypothesis. The observed decline in serum Hp in the treatment group in our study agrees with the result of a previous study, which reported a decreased concentration of serum Hp in sheep during an outbreak of severe haemolytic anaemia due to natural *M. ovis* infection (Faraj & Kamal 2017). This report provided evidence of the involvement of APP in the pathogenesis of *M. ovis* infection in small ruminants. Physiologically, the Hp molecule binds free haemoglobin (Hb), forming a Hp-Hb complex, which prevents the formation of oxygen radicals and oxidative tissue damage during haemolysis (Smith & Roberts 1994). Also similar to our observation in this study concerning Hp levels, the serum Hp, which is a potential indicator of haemolysis, shows a decreasing trend during haemolytic episodes (Jain *et al*. 2011). Interestingly, the Hp-Hb complex exerts bacteriostatic effects by making iron unavailable for bacterial cell metabolism (Ceciliani *et al*. 2012) and modulates inflammation/immunity by inhibiting T helper 2 cells (Th-2) response and mast cell proliferation (Murata *et al*. 2004). Even though Hp is a nonspecific marker of anaemia, it is essential to further explore its role as a potential marker of subclinical or acute haemolytic anaemia during *M. ovis* infection in small ruminants under field conditions.

The increased levels of SAA proteins in the treatment group indicate a positive acute-phase response to *M. ovis* infection. Korman *et al*. (2012) have previously demonstrated significant expression of SAA proteins in *Mycoplasma haemofelis* infected cats. The endogenous production of SAA proteins in the liver occurs in response to specific chemokines secreted by activated leucocytes during infection (Uhlar & Whitehead 1999). The concentration of SAA proteins may increase exponentially during systemic inflammation (Shah *et al*. 2006). For instance, an elevated concentration of SAA is associated with the diagnosis of clinical mastitis in dairy cows ([Bibr b14-tlsr_35-3-319][Bibr b41-tlsr_35-3-319]; Korman *et al*. 2012). Even though APPs are nonspecific biomarkers, they represent appropriate analytes for assessing animal health and the nutritional state of animals (Gruys *et al*. 2005). Thus, the assay of APPs is an instrument for detecting tissue injury and inflammation and evaluating the prognosis and treatment progress within the clinical environment (Thompson *et al*. 1992). However, despite considerable research efforts showcasing the potential of the APPs, many characteristics of the APPs in small ruminant haemotropic mycoplasmosis have yet to be expounded.

Contrary to the null hypothesis, the reproductive hormonal assay revealed different oestrogen and progesterone secretion patterns in the treatment and the control groups. The reduction in serum oestrogen with a simultaneous significant increase in serum progesterone in the treatment group suggests a metoestrus phase, which implies a state of pseudopregnancy in infected mice (Entrican & Wheelhouse 2006; Frandson *et al*. 2013). According to Tsai and O’Malley (1994), oestrogen and progesterone produced mainly in the ovaries regulate reproductive events such as cellular proliferation, differentiation, development, apoptosis and inflammation. Infectious diseases are known to interfere with the reproductive physiology of small ruminants (Baird & Fontaine 2007; Khanum *et al*. 2008) by causing irregular cycling activities, infertility, congenital malformations, premature delivery and abortions (Schimmer *et al*. 2011; Wernike *et al*. 2013; World Organisation on Animal Health (WOAH) 2018). Haemorrhagic septicaemia, caseous lymphadenitis, and brucellosis profoundly interfere with the reproductive physiology of small ruminants (Baird & Fontaine 2007; Jesse *et al*. 2016). Several studies have attempted to explain how certain diseases interfere with hormonal balance, conception and the maintenance of pregnancy in domestic animals. According to Moore and Moger (1991), disease pathogens induce the release of inflammatory mediators such as cytokines and prostaglandin (PG)-F2α, which are responsible for lowering fertility by suppressing steroidogenesis. Suppression of female reproductive hormones in sick goats stimulates the phagocytic activity of neutrophils (Maynard & Downes 2019). However, other studies proposed that disease processes cause the production of lesions in the anterior pituitary gland and interfere with GnRH, which is an important factor that regulates the synthesis and secretion of gonadotropins (Jesse *et al*. 2017).

The regulatory roles of FSH and LH on ovulation and steroidogenesis could be interrupted by bacterial infections, especially members of the *Pasteurella* group (Maqbool *et al*. 2022). For instance, the oral administration of *P. multocida* type B: 2 and its lipopolysaccharides altered serum progesterone and oestrogen concentrations (Jesse *et al*. 2014). Increased serum progesterone hormone levels were also observed in female goats challenged with *M. haemolytica* or its LPS endotoxin during the follicular phase of the oestrous cycle (Kamarulrizal *et al*. 2022). Furthermore, previous studies have also documented increased plasma oestrogen levels in goats and mice experimentally inoculated with *C. pseudotuberculosis* (Khuder *et al*. 2012; Jesse *et al*. 2014; Othman *et al*. 2014). Elevated serum oestrogen in goats infected with *C. pseudotuberculosis* has been linked to cellular injury in the ovaries and pituitary gland (Jesse, Latif, *et al*. 2015).

The gross lesions seen in the ovary of *M. ovis*-infected mice have not been reported in relation to haemoplasma infection. The observed changes in the ovaries of *M. ovis*-infected mice are like previous studies on reproductive hormonal responses to *P. multocida* B2 in female mice and buffalo heifers (Jesse *et al*. 2017; Ibrahim *et al*. 2018). Leukocytic infiltration, congestion and degenerations of ovarian follicles were observed previously in mice challenged with *C. pseudotuberculosis* and its exotoxin (Khuder *et al*. 2012). Degeneration, necrosis, leucocytic infiltration and generalised congestion of reproductive organs were also observed during experimental infection of *C. pseudotuberculosis* in goats (Jesse *et al*. 2011; Latif *et al*. 2015; 2017).

The pathological changes in the ovary of mice due to experimental infection of *M. ovis* may potentially influence folliculogenesis and drive hormonal imbalances. The increased activity of white blood cells observed in the spleen and the activation of Kupffer cells in the liver indicates an active response to infection due to increased phagocytosis of infected red blood cells. This finding may explain the decreased PCV observed at the peak of parasitaemia and corroborates the changes in acute phase reactants. Hp and SAA proteins are immune opsonins and binding proteins which facilitate the removal of *M. ovis*-infected red blood cells by phagocytosis in the liver and spleen. The hepatic lesions seen in mice due to increased phagocytosis of infected red blood cells by the Kupffer cells (John & Invermay 1990; Philbey *et al*. 2006) have been described in small ruminants (Mason *et al*. 1981; Fitzpatrick *et al*. 1998; Philbey *et al*. 2006). The proliferative glomerulonephritis in the kidneys of *M. ovis*-infected mice was previously described in mice (Kanabathy & Nachiar 2004) and sheep (Sutton 1978). The observed cellular changes in mouse kidneys could be due to glomeruli damage caused by immune complex formation during the phagocytosis of *M. ovis*-infected red blood cells.

## CONCLUSIONS

The disturbance in progesterone and ovarian pathology recorded here is a novel aspect of this study that highlights the potential role of haemotropic *M. ovis* in reproductive disorders. The observed changes in biomarkers and cellular reactions following *M. ovis* infection in the mouse may be further advanced in sheep and goats to consolidate our findings. The slight differences in the incubation period and progression of parasitaemia between the mouse and sheep would suggest a possible difference in the pattern and degree of acute-phase reaction. Yet, such minor differences would not impair the translational use of the preliminary data reported here. Finally, it is worth noting that the actual role of inflammatory proteins in *M. ovis* infection is a new concept that requires further investigations, including consideration of potential molecular crosstalk between inflammatory mediators and the reproductive system.

## Figures and Tables

**Table 1:** Mean values of serum biomarkers of mice responding to *M. ovis* infection.

Biomarkers	Control	Treatment	*t*-test	*P*
Oestrogen	17.43 ± 4.48^a^	10.38 ± 5.07^b^	2.330	0.048
Progesterone	15.92 ± 4.20^a^	27.37 ± 2.17^b^	5.415	0.010
Haptoglobin	18.16 ± 1.98^a^	8.72 ± 1.49^b^	8.525	<0.001
Serum amyloid	11.59 ± 2.15^a^	16.80 ± 2.71^b^	3.362	0.010

*Note*: Means with different superscripts (^a,b^) differed significantly (*P <* 0.05).
